# Vision-related quality of life in patients with glaucoma: the role of illness perceptions

**DOI:** 10.1186/s12955-022-01979-x

**Published:** 2022-05-12

**Authors:** Qiqi Zhang, Wenzhe Zhou, Di Song, Yanqian Xie, Hao Lin, Youping Liang, Yanhan Ren, Yuanbo Liang, Yanyan Chen

**Affiliations:** 1 School of Optometry and Ophthalmology, Wenzhou Medical University, Wenzhou, Zhejiang China; 2 The Eye Hospital, Wenzhou Medical University, 270 Xueyuan West Road, Lucheng District, Wenzhou, 325000 Zhejiang Province China; 3 Huzhou First People’s Hospital, Huzhou, Zhejiang China; 4 Chicago Medical School, Rosalind Franklin University of Medicine and Science, North Chicago, IL USA

**Keywords:** Glaucoma, Vision-related quality of life, Illness perceptions, Common sense model

## Abstract

**Purpose:**

To explore the predictive effects of illness perceptions on vision-related quality of life (VRQoL) in Chinese glaucoma patients.

**Methods:**

In this cross-sectional study, 97 patients with glaucoma completed the brief illness perception questionnaire (BIPQ), the glaucoma quality of life-15 (GQL-15) questionnaire, and a questionnaire regarding sociodemographic and clinical information. A correlation analysis and hierarchical linear regression analysis were performed.

**Results:**

The BIPQ total score was positively correlated with the total score of the GQL-15 questionnaire and the scores of its four dimensions. Chronic comorbidities, the type of glaucoma, the best-corrected visual acuity (BCVA), the mean defect (MD) of visual field in the better eye, and identity in the BIPQ were critical predictors of VRQoL. Illness perceptions independently accounted for 7.8% of the variance in the VRQoL of glaucoma patients.

**Conclusions:**

Patients with stronger illness perceptions and those who perceive themselves as having more glaucoma symptoms are likely to experience worse VRQoL. Illness perceptions in glaucoma patients deserve clinical attention, and further studies are needed to examine whether cognitive interventions targeting illness perceptions can improve VRQoL.

**Supplementary Information:**

The online version contains supplementary material available at 10.1186/s12955-022-01979-x.

## Plain English summary

Glaucoma is the leading cause of irreversible blindness worldwide. Visual acuity loss and visual field defects caused by pathological high intraocular pressure are the main features of the disease. The quality of life of patients with glaucoma is often affected by the impairment of visual function. Illness perceptions (how patients perceive their disease) have been shown to be closely related to self-reported quality of life in other chronic diseases and seem to affect disease progression and prognosis. In this study, we explored the impact of illness perceptions on vision-related quality of life in Chinese glaucoma patients. Perception questionnaires were used to record patients’ self-reported outcomes. The results of this study indicate that patients with more self-perceived glaucoma symptoms tend to have a lower level of vision-related quality of life. This study highlights the importance of illness perceptions in glaucoma patients and provides a theoretical basis for improving vision-related quality of life in glaucoma patients according to psychological cognitive interventions.

## Background

As the most frequent cause of irreversible blindness [1], glaucoma has become one of the major diseases affecting national visual health and quality of life (QoL) in China [2, 3]. Due to rapid ageing trends [4], it is estimated that the total number of glaucoma cases in China will be 25.16 million by 2050, which will undoubtedly put a heavy burden on the social economy and healthcare systems [5].

In clinical practice, glaucoma patients have lower QoL than the general population, and many studies have reported that glaucoma patients with worse vision acuity (VA) and greater visual field (VF) loss have poorer QoL [6–8]. In addition, limited vision-related movement, the very large economic and psychological burden of lifelong treatment and follow-up were also important factors contributing to the impairment of QoL [5, 9, 10]. Poor QoL affects patients' confidence in glaucoma treatment and incentives for self-management, resulting in a vicious cycle. Therefore, maintaining visual function and related QoL is a critical goal of glaucoma treatment.

Many studies have confirmed that illness perceptions play an important role in explaining chronic disease patients' QoL [11]. For instance, high levels of illness perceptions are linked to poor health-related QoL in patients with breast cancer [12]. It has also been reported that different aspects of illness perceptions account for varying degrees of variance in both physical and mental QoL [13]. Most of these studies have obtained perception measurements via patient-reported outcome instruments. Positive illness perceptions are beneficial in the context of disease prevention, treatment and prognosis [14, 15]. Illness perception is a psychological concept based on the common sense model (CSM) of self-regulation proposed by Leventhal et al. [16], which emphasizes patients' lay perspective towards disease. In the CSM, patients' cognitive representations of illness were conceptualized into the following components: identity (the symptoms experienced by the individual), timeline (the duration of the illness), consequence (the extent to which the illness affects daily life), course (the perceived causal antecedents of the illness), perceived control (individuals' belief in the controllability and curability of the illness) and coherence (individuals’ understanding of their illness). Emotional representations (individuals' emotional responses to the illness) were also included as an additional component of the model [17]. Generally, patients' subjective views may lead to changes in their coping strategies and psychological states and then exert direct or indirect influences on disease progression through a self-regulatory system [18]. Some research evidence indicates that targeted interventions aimed at improving patients' illness perceptions can also have significant implications for their clinical outcomes [19–22]. Accordingly, as a typical chronic psychosomatic disease in the field of ophthalmology, glaucoma deserves attention in the context of illness perceptions of patients.

To the best of our knowledge, few studies have investigated the association between illness perceptions and QoL in patients with glaucoma. Therefore, the aim of this study was to explore the predictive value of illness perceptions for vision-related quality of life (VRQoL) in Chinese glaucoma patients, hoping to understand how patients with glaucoma make sense of their illness and to shed new light on approaches to increase VRQoL.

## Methods

### Participants and setting

This cross-sectional survey was conducted in an ophthalmic hospital in Zhejiang Province, southern China, between September 2020 and March 2021. A total of 97 glaucoma patients were recruited. The inclusion criteria were as follows: (1) diagnosed with glaucoma, including primary and secondary glaucoma; (2) used topical ocular medications to reduce intraocular pressure (IOP) for at least 2 weeks; and (3) aged 18 years or older. The exclusion criteria were as follows: (1) patients with any other ocular diseases that might affect the retinal nerve fibre layer or the VF; (2) patients with severe diseases of the heart, liver, kidney or nervous system; and (3) patients diagnosed with a psychiatric disorder or severe cognitive impairment.

### Data collection

Individual face-to-face interviews were conducted by a trained interviewer. Patients were asked to complete the questionnaire by themselves. For those who had difficulty reading or writing, the questionnaire was completed by the interviewer based on the patient's oral answers. All questionnaires were immediately collected and reviewed to confirm consistency and completeness.

The ophthalmological examination results of VA and VF were obtained from the hospital electronic medical system and were recorded by trained ophthalmologists at the clinical research and examination centre of the hospital.

### Measures

#### Sociodemographic and clinical data

The information provided by patients included the following: age, gender, educational level, monthly per capita income (calculated as family income divided by the number of family members), chronic comorbidities (including hypertension, diabetes mellitus, hyperlipidaemia, cardiovascular disease and chronic pulmonary/kidney/liver disease), smoking history (defined as current smoking or smoking cessation), type of glaucoma, duration of glaucoma, glaucoma family history, number of antiglaucoma medications used, the mean defect (MD) of VF, and the best-corrected visual acuity (BCVA, determined using the Snellen visual acuity chart, and converted to a logarithm of the minimum angle of resolution score (logMAR)). VF testing was performed with the Humphrey Field Analyzer 750i (Carl Zeiss Meditec, Inc., Dublin, CA, USA) using the Swedish Interactive Threshold Algorithm (SITA) standard central 24-2 program. The reliability parameters of fixation losses < 20% and false positives/negatives < 15% were used.

#### The brief illness perception questionnaire (BIPQ)

The Chinese version of the BIPQ (Broadbent et al. [23]) was used to measure 3 dimensions of illness perception: illness comprehensibility, illness cognitive representations and emotional representations. A previous study reported acceptable reliability and validity of this scale among a Chinese glaucoma population [24]. The scale consists of 8 scoring items. Items 1 to 8 correspond to consequences, timeline, amount of perceived personal control, treatment control, identity (symptoms), illness concern, illness coherence and emotional representation. Each item is rated on a scale from 0 to 10. A total score is calculated by summing all the item scores (items 3, 4, and 7 are reverse scored) with a potential range between 0 and 80, with higher scores indicating a more threatening perception of illness.

#### The glaucoma quality of life-15 (GQL-15) questionnaire

The GQL-15 questionnaire was developed by Nelson et al. [25] and translated into Chinese by Zhou et al. to measure VRQoL in patients with glaucoma. The Chinese version of the GQL-15 (GQL-15-CHI) showed psychometric properties comparable to the original English version, with an overall Cronbach's alpha of 0.96 [26]. The scale contains 15 items covering 4 dimensions: central and near vision (two items), peripheral vision (six items), glare and dark adaptation (six items), and outdoor mobility (one item). Responses are provided on a Likert-type scale from 1 (no difficulty) to 5 (severe difficulty), with an additional option of “not applicable” included. The total possible score ranges from 15 to 75. For the scores of each dimension, the raw scores of these items are first transformed into a 0–100 scale (1, 2, 3, 4 and 5 were converted into 0, 25, 50, 75 and 100, respectively), and then an average score was calculated by summing up the item scores in that domain and dividing by the total number of items, with higher scores indicating worse functioning and poorer VRQoL.

### Ethical considerations

This study complied with the Declaration of Helsinki and was approved by the Ethics Committee of the Affiliated Ophthalmology and Optometry Hospital of Wenzhou Medical University (Project Number: 2020-180-K-163-01). Patients were informed that the purpose of the study and that any details of their responses would be kept confidential. All enrolled patients signed a written informed consent form.

### Power calculation

For the hierarchical linear regression analysis approach in this study, a sample size of 97 achieves 84% power using a two-sided F test with a significance level of 0.05 and a conventional effect size of 0.25.

### Statistical analyses

In the descriptive analysis, scale scores and continuous variables of demographic data are summarized as the mean (standard deviation, SD), whereas categorical variables are shown as the frequency and percentage. T-tests and one-way ANOVA were performed to evaluate differences between and among independent groups. Pearson's correlation analysis and canonical correlation analysis (CCA) were used to determine the association between each item of the BIPQ and GQL-15 domain scores. Multiple comparisons were performed by Bonferroni post-hoc test to evaluate between-group differences in mean scores. Furthermore, hierarchical linear regression was conducted to explore the effect of illness perceptions on VRQoL after controlling for sociodemographic variables and disease characteristics. Only variables that had a significant effect on VRQoL were candidates for inclusion in the regression analysis. The level of statistical significance was set at *P* < 0.05 for all analyses. All statistical analyses were performed using IBM SPSS software version 25.0 (SPSS Inc., Chicago, IL, United States).

## Results

### Sociodemographic and clinical characteristics

A total of 105 eligible patients were initially recruited in the study. The data from 8 participants were excluded from analysis because the participants failed to cooperate with the eye examination and their VF test results were unreliable. Of the remaining 97 subjects, 44 (45.4%) were male and 53 (54.6%) were female, and the average age was 54.87 (SD = 14.06, range 24–80) years. The sociodemographic and clinical characteristics of the sample are shown in Table 1. Most of the patients (72.2%) were diagnosed with primary glaucoma, and nearly half of them (47.4%) were treated with three kinds of topical drugs to lower IOP. The average logMAR BCVA in the better eye and worse eye were 0.30 (SD = 0.71) and 0.84 (SD = 1.02), respectively. The average MD was − 10.85 (SD = 9.85) dB in the better eye and − 20.48 (SD = 10.30) dB in the worse eye. The most frequent chronic comorbidity observed in participants was hypertension followed by diabetes.

**Table 1:** Sociodemographic and clinical characteristics (n = 97)

Variables	n (%)	BIPQ Scores Mean (SD)	*P* value	GQL-15 Scores Mean (SD)	*P* value
Gender			0.985		0.001**
Male	44 (45.4)	50.32 (8.66)		32.41 (14.41)	
Female	53 (54.6)	50.28 (9.03)		23.85 (8.40)	
Educational level			0.963		0.933
Illiterate	16 (16.5)	51.00 (8.35)		26.13 (10.31)	
Elementary school	33 (34.0)	50.48 (10.30)		27.33 (11.14)	
Middle school	22 (22.7)	50.68 (8.67)		27.55 (14.24)	
High school	8 (8.2)	50.25 (3.37)		30.38 (14.69)	
University or above	18 (18.6)	48.89 (8.82)		28.94 (13.07)	
Monthly income (RMB)			0.228		0.158
< 1500	10 (10.3)	55.30 (8.14)		34.40 (9.48)	
1500~	26 (26.8)	48.42 (8.97)		25.62 (10.32)	
3000~	27 (27.8)	51.56 (8.73)		24.96 (9.24)	
5000~	23 (23.7)	49.78 (8.13)		30.78 (14.79)	
≥ 10,000	11 (11.3)	48.18 (9.91)		27.09 (16.96)	
Type of glaucoma			0.000***		0.018*
Primary glaucoma	70 (72.2)	48.37 (7.76)		25.91 (11.19)	
Secondary glaucoma	27 (27.8)	55.30 (9.57)		32.44 (13.72)	
Duration of glaucoma			0.606		0.207
< 3 months	22 (22.7)	48.18 (10.90)		24.27 (11.74)	
3 months~	22 (22.7)	50.18 (9.65)		25.14 (9.49)	
1 year~	37 (38.1)	50.89 (7.42)		30.92 (14.30)	
5 years~	7 (7.2)	54.14 (8.49)		26.14 (11.45)	
≥ 10 years	9 (9.3)	50.33 (6.95)		30.67 (8.31)	
Family history			0.596		0.937
Yes	11 (11.3)	51.64 (9.48)		27.45 (12.86)	
No	86 (88.7)	50.13 (8.78)		27.77 (12.23)	
Number of antiglaucoma medication used			0.287		0.847
1	10 (10.3)	53.80 (8.35)		27.90 (12.68)	
2	28 (28.9)	49.57 (10.38)		28.14 (10.60)	
3	46 (47.4)	49.15 (7.41)		26.67 (12.74)	
4	10 (10.3)	54.60 (8.62)		29.30 (13.86)	
≥ 5	3 (3.1)	48.67 (14.15)		34.33 (17.90)	
Chronic comorbidities			0.021*		0.001**
Yes	40 (41.2)	52.75 (8.25)		32.83 (14.68)	
No	57 (58.8)	48.58 (8.87)		24.16 (8.66)	
Smoking history			0.559		0.004**
Yes	27 (27.8)	51.15 (8.42)		34.78 (15.58)	
No	70 (72.2)	49.97 (9.01)		25.01 (9.48)	

*P < 0.05; **P < 0.01; *** *P* < 0.001

### BIPQ and GQL-15 scores and Pearson's correlation analysis

The BIPQ and GQL-15 scores for different categories of variables are presented in Table 1. Of all the variables, both total scores showed significant differences between groups with regard to the type of glaucoma and chronic comorbidities. According to the GQL-15 scores, gender and smoking history also showed significant differences between groups. There was no significant difference between groups for the other variables.

Correlation analyses showed that the BIPQ total score was positively correlated with the total GQL-15 and its four dimensions. This finding indicates that a more negative perception of illness was related to worse VRQoL. The specific values of the subscales and correlation coefficients are shown in Table 2. Specifically, the score assigned to illness concern (item 6 = 9.79 ± 0.88) was the highest among the items of BIPQ, while treatment control (item 4 = 3.64 ± 2.20) was the lowest. The subscale scores of the GQL-15 indicated that the patients generally had worse function in the glare and dark adaptation domain (25.08 ± 23.26) followed by the central vision and near vision domain (23.71 ± 27.75) and relatively good function in outdoor mobility (14.95 ± 28.10). Except for scores on the timeline, treatment control, illness concern, illness coherence, and emotional representation items of the BIPQ, which had no significant relationship with the GQL-15, consequences, personal control, and identity were all significantly positively correlated with the GQL-15.

**Table 2:** BIPQ and GQL-15 scores: correlation coefficient values (Pearson r) between BIPQ and GQL-15

	Scores Mean (SD)	BIPQ	BIPQ1	BIPQ2	BIPQ3	BIPQ4	BIPQ5	BIPQ6	BIPQ7	BIPQ8	GQL-15	GQL-1501	GQL-1502	GQL-1503	GQL-1504
BIPQ	50.30 (8.82)	1.000													
BIPQ1	7.20 (2.81)	0.614**	1.000												
BIPQ2	6.38 (3.16)	0.380**	0.069	1.000											
BIPQ3	5.03 (2.24)	0.675**	0.249^*^	0.385**	1.000										
BIPQ4	3.64 (2.20)	0.445**	0.047	0.047	0.433**	1.000									
BIPQ5	5.97 (2.81)	0.494**	0.378**	0.174	0.080	− 0.131	1.000								
BIPQ6	9.79 (0.88)	− 0.044	0.067	− 0.092	− 0.209^*^	0.134	− 0.134	1.000							
BIPQ7	6.43 (3.19)	0.101	− 0.179	− 0.477**	0.108	0.113	− 0.217^*^	− 0.165	1.000						
BIPQ8	5.86 (2.73)	0.636**	0.430**	0.055	0.170	0.133	0.314**	− 0.034	− 0.008	1.000					
GQL-15	27.73 (12.23)	0.398**	0.476**	0.094	0.224^*^	0.098	0.400**	− 0.001	− 0.071	0.095	1.000				
GQL-1501	23.71 (27.75)	0.264**	0.362**	0.093	0.261**	0.126	0.212^*^	− 0.081	− 0.066	− 0.058	0.803**	1.000			
GQL-1502	19.80 (21.21)	0.349**	0.416**	0.041	0.147	0.069	0.371**	0.019	0.002	0.084	0.919**	0.641**	1.000		
GQL-1503	25.08 (23.26)	0.420**	0.467**	0.121	0.230^*^	0.073	0.435**	− 0.023	− 0.102	0.169	0.924**	0.694**	0.746**	1.000	
GQL-1504	14.95 (28.10)	0.314**	0.374**	0.111	0.162	0.096	0.303**	− 0.096	− 0.056	0.073	0.785**	0.751**	0.701**	0.664**	1.000

BIPQ1: consequences, BIPQ2: timeline, BIPQ3: personal control, BIPQ4: treatment control, BIPQ5: identity, BIPQ6: illness concern, BIPQ7: illness coherence BIPQ8: emotional representation; GQL-1501: central and near vision, GQL-1502: peripheral vision, GQL-1503: glare and dark adaptation, GQL-1504: outdoor mobility

^*^P < 0.05; **P < 0.01

Furthermore, there were positive correlations between logMAR BCVA in the better eye (r = 0.579, *P* < 0.01) and the worse eye (r = 0.459, *P* < 0.01) and overall GQL-15, while MD was negatively correlated with GQL-15 (MD in the better eye r = − 0.644, *P* < 0.01; MD in the worse eye r = − 0.249, *P* < 0.05).

### CCA

CCA was introduced to study the correlation of two sets of variables [27]. The first set of variables consisted of 4 dimensions in the GQL-15 questionnaire, Y1–Y4, which correspond to GQL1501, GQL1502, GQL1503, and GQL1504. The second set contained 8 items in the BIPQ, X1–X8, which correspond to BIPQ1, BIPQ2, BIPQ3, BIPQ4, BIPQ5, BIPQ6, BIPQ7, and BIPQ8. Two multivariate variables, V and U, were extracted from the above two sets of variables. The results showed that there were four pairs of canonical correlation variables (V1–V4 and U1–U4). We focused on the first pair of canonical variables (V1 and U1), which had a significant correlation (*P* < 0.05) and captured the largest proportion of the variation shared between the BIPQ and GQL-15; the correlation coefficient was 0.586. The standardized canonical correlation coefficients and canonical loading coefficients of sets V1 and U1 are shown in Table 3.

**Table 3:** Canonical correlation coefficients for GQL-15 and BIPQ scores

	Variables	Standardized canonical correlation coefficients	Canonical loading coefficients
V1	Y1	− 0.004	− 0.745
	Y2	− 0.314	− 0.884
	Y3	− 0.652	− 0.966
	Y4	− 0.116	− 0.772
U1	X1	− 0.703	− 0.818
	X2	− 0.068	− 0.179
	X3	− 0.083	− 0.368
	X4	− 0.165	− 0.138
	X5	− 0.601	− 0.743
	X6	− 0.013	0.035
	X7	− 0.138	0.124
	X8	0.282	− 0.247

Y1: GQL-1501 (central and near vision), Y2: GQL-1502 (peripheral vision), Y3: GQL-1503 (glare and dark adaptation), Y4: GQL-1504 (outdoor mobility); X1: BIPQ1 (consequences), X2: BIPQ2 (timeline), X3: BIPQ3 (personal control), X4: BIPQ4 (treatment control), X5: BIPQ5 (identity), X6: BIPQ6 (illness concern), X7: BIPQ7 (illness coherence), X8: BIPQ8 (emotional representation)

### Hierarchical linear regression analysis

As shown in Table 4, only variables with a *P* value < 0.05 in univariate analysis were entered into the hierarchical linear regression model. In Model 1, demographic covariates such as gender, chronic comorbidities, and smoking history were evaluated as potential confounders. After controlling for the above three variables, chronic comorbidities, type of glaucoma, logMAR BCVA in the better eye, and MD in the better eye still exerted significant effects on VRQoL in Model 2, and the explanatory power of the model improved by 38.7% (ΔR^2^ = 0.387). Model 3 included each item of the BIPQ, and the standardized regression coefficient of identity was positive and statistically significant, indicating that higher identity beliefs predict lower VRQoL. This model explained 70.1% of the total variance in VRQoL, and illness perceptions independently accounted for 7.8% of the variance in VRQoL. All models achieved statistical significance.

**Table 4:** Predictors of VRQoL in patients with glaucoma (n = 97)

Variables	Model 1		Model 2		Model 3	
	β	t	β	t	β	t
Gender	− 0.157	− 1.261	− 0.162	− 1.787	− 0.141	− 1.457
Chronic comorbidities	0.297	3.226**	0.201	2.974**	0.225	3.153**
Smoking history	0.213	1.717	0.035	0.374	0.047	0.497
Type of glaucoma			0.248	3.728***	0.194	2.837**
MD in the better eye			− 0.407	− 3.728***	− 0.383	− 3.576**
MD in the worse eye			− 0.014	− 0.165	0.001	0.008
LogMAR in the better eye			0.278	3.307**	0.239	2.893**
LogMAR in the worse eye			− 0.008	− 0.088	− 0.042	− 0.491
BIPQ1					0.056	0.630
BIPQ2					− 0.136	− 1.559
BIPQ3					0.111	1.231
BIPQ4					0.017	0.216
BIPQ5					0.259	3.549**
BIPQ6					0.070	0.998
BIPQ7					− 0.057	− 0.607
BIPQ8					− 0.046	− 0.585
R^2^	0.235		0.622		0.701	
Adjusted R^2^	0.211		0.588		0.641	
ΔR^2^	0.235		0.387		0.078	
F	9.548***		18.136***		11.708***	

BIPQ1: consequences, BIPQ2: timeline, BIPQ3: personal control, BIPQ4: treatment control, BIPQ5: identity, BIPQ6: illness concern, BIPQ7: illness coherence BIPQ8: emotional representation

^*^P < 0.05; **P < 0.01; ***P < 0.001

In summary, the results from the hierarchical linear regression model demonstrated that poor VA, severe VF defects in the better eye, and a strong identity are closely related to poor VRQoL in patients with glaucoma.

## Discussion

This was the first study to investigate the relationship between illness perceptions and VRQoL in a Chinese glaucoma population. The hierarchical linear regression model explained up to 70.1% of the variance in VRQoL, and the clinical variables accounted for the greatest variance in the model. Chronic comorbidities, type of glaucoma, BCVA and MD in the better eye, and identity (symptoms) of illness perceptions are the critical predictors of VRQoL according to our data. These findings suggest that primary glaucoma patients without chronic disease have better BCVA and fewer VF defects in their better eye, while those who perceive fewer symptoms attributable to glaucoma are likely to experience higher VRQoL.

Our study highlights the specific contributions of illness perceptions to glaucoma patients' VRQoL after controlling for demographic and clinical variables. Illness perception has been considered an important psychological factor affecting adherence and QoL in patients with some chronic diseases, such as cancer [28], chronic kidney disease [29], diabetes [30], cardiovascular disease [31] and chronic obstructive pulmonary disease (COPD) [32]. Specifically, previous studies have reported that higher identity was a strong predictor of outcomes, predicting lower QoL in coronary heart disease (CHD) patients [15], which is consistent with our findings. However, existing studies in the field of glaucoma have tended to analyse the relationship between illness perceptions and adherence or between adherence and QoL; thus, their results only briefly mention a univariate association between illness perceptions and QoL without further exploration [33–35]. Studies have suggested that identity has a significant impact on medication adherence [36] and that glaucoma patients with better adherence tend to report a better perceived QoL [33]; given these findings and the results of the present study, we speculate that interventions targeted at identity may improve QoL. A randomized controlled trial (RCT) revealed a reduction in identity scores, corresponding to significantly fewer complaints of symptoms in the cognitive behavioural therapy (CBT) intervention group [37]. Another RCT that used psychological family-based interventions for patients with type 2 diabetes also reported statistically significant improvements in health outcomes, including glycaemic control (a decrease in glycated haemoglobin) and diabetes identity (a reduction in perceived symptom burdens) [38]. These examples imply that identity, as one of the core cognitive dimensions in CSM, is modifiable through psychological interventions such as CBT. Therefore, subsequent studies could try to examine whether cognitive interventions are equally effective in glaucoma patients.

The total BIPQ score in our sample population was higher than that among patients with diabetes and COPD [39–41]. Glaucoma patients are more likely to perceive the threat of illness, possibly because the impairment of visual function caused by disease progression can severely affect the patient's mobility and cause them to lose independence. We ranked the item scores from highest to lowest and compared the ranking of each item in our study with that in other studies. The results showed that the “consequence” and “identity” items ranked higher in our study than in other studies of glaucoma populations in other countries [42–45]. The rank of the “concern” item in our study was similar to that in studies of Singaporean (of Chinese descent) populations [44, 45], but higher than that in some studies in European countries [42, 43]. In addition, the rank of the “timeline” and “treatment control” items were lower in our study than in other studies. On the one hand, this difference might partly be attributed to diverse cultural backgrounds and inclusion criteria. On the other hand, the low timeline and treatment control scores indicate that most Chinese patients are not conscious of the chronic and permanent characteristics of glaucoma, are less likely to recognize the effectiveness of treatment and more likely to consider the disease hard to control. We speculate that these differences may exist because glaucoma is not as common as other chronic diseases, such as diabetes and hypertension, and because public awareness of this eye disease is low. In addition, our sample mainly comprised elderly individuals with a primary educational level, whose capability and channels to receive reliable information about glaucoma are limited. When patients have an insufficient understanding of the disease (including difficulty in recognizing symptoms and unawareness of the consequences of the disease), the visual field loss caused by glaucoma, which is irreversible even after treatment, is likely to increase patient concern over the illness, thus leading to high scores for illness consequence, identity and concern on the BIPQ. Our CCA results confirmed the significant positive correlations between illness perceptions and VRQoL, especially the effects of consequence and identity on the glare and dark adaptation dimensions. The results also provide new ideas for improving the QoL of glaucoma patients; perhaps helping them correctly identify the characteristics and consequences of glaucoma could be an important starting point for interventions. Of course, intervention studies are required to confirm the validity and feasibility of such measures.

One of the important results of our study was that clinical parameters such as VA and VF could explain the most variance in VRQoL in the model. LogMAR BCVA and MD of the better eye were independent predictors of VRQoL. This finding suggests that the condition of the better eye was more important for glaucoma patients' VRQoL than that of the worse eye, which is in line with previous studies [6, 8, 46]. Epidemiological studies show that diagnosis and treatment are often delayed due to the relatively asymptomatic damage caused by glaucoma in the early stages, and the majority of patients do not realize they have glaucoma until their condition deteriorates to the point where their bilateral vision is severely impaired. Therefore, early identification and treatment are the key to maintaining VRQoL [10]. Nevertheless, ophthalmic examinations, surgeries, medications, and other treatments aimed at reducing IOP are usually concentrated on the worse eye. The results of our study emphasize the importance of monitoring the visual function of the better eye as early as possible. Noticeably, chronic comorbidities and the type of glaucoma were identified as predictors affecting VRQoL in our sample. This finding indicates that patients with other chronic comorbidities and patients with secondary glaucoma have a poorer VRQoL. However, a study of Chinese glaucoma patients conducted by Zhou et al. [46] concluded that VRQoL was associated with economic burden, VA, VF, number of glaucoma surgeries and depression and did not mention the two factors we identified. This difference could be explained by the varied types of grouping and the different proportions of patients in each group; in addition, chronic comorbidities were not taken into account in their study. Unlike primary glaucoma, which has a covert onset, most of the secondary glaucoma patients included in our study had a specific cause of elevated IOP, such as trauma, certain medications (e.g., corticosteroids) or other diseases (e.g., tumour). We speculate that in addition to the primary disease, patients' illness perceptions over time that are potentially amenable to dynamic change related to the complex treatment experience, along with the increase in BIPQ scores, have a direct or indirect impact on VRQoL [47, 48]. We used the bootstrap approach based on the Preacher and Hayes [49] procedure to conduct an additional mediation analysis and used the PROCESS macro provided by SPSS (the number of bootstrap samples was 5000) for the bias-corrected bootstrap confidence interval, which is considered to be more powerful than the Sobel test for smaller samples [50, 51]. The BIPQ measuring illness perceptions was entered as a potential mediator in two simple mediation models with VRQoL as the dependent variable, and chronic comorbidities and the type of glaucoma as the independent variables. The result showed that illness perceptions partially mediated the relationship between chronic comorbidities and VRQoL, whereas completely mediated the relationship between the type of glaucoma and VRQoL (see Additional file 1). This result indicates that conducting a cognitive assessment of illness (such as illness perceptions) may be necessary for patients with different illness backgrounds and treatment experiences, as illness perceptions play an important role in determining patients’ VRQoL. Some studies on other diseases have also found a significant mediating role of illness perceptions in affecting patients' physical and mental health [52, 53]. Perhaps follow-up longitudinal studies (or qualitative interviews) can help us better understand and explain the reasons behind this mediating effect. However, the above findings seem to lend credence to our result that patients with chronic comorbidities and secondary glaucoma are anticipated to have worse VRQoL; for this reason, more attention and support should be given to these patients.

As a cross-sectional study, this study inevitably has some limitations. We cannot confirm the specific causal relationships between predictors and outcomes. In addition, the study population was recruited from a single medical centre, and the sample size was relatively small, which limits our power and generalizability. Further longitudinal studies or RCTs are necessary. We also expect that future studies will include more potential psychological variables that might influence patients' behaviour and QoL to deeply explore the functional routes of each factor and provide more empirical evidence for clinical psychological cognitive interventions.

## Conclusion

Illness perceptions have a stable effect on VRQoL in Chinese glaucoma patients, and patients with stronger illness perceptions have poorer VRQoL. Moreover, among illness perceptions, identity was identified as an important predictor of VRQoL after controlling for sociodemographic and clinical variables. The more symptoms patients perceived related to their glaucoma, the worse their VRQoL. Our study highlights the importance of paying attention to the subjective views of glaucoma patients and their perceptions of illness in clinical practice, and our results provide a theoretical basis for improving the VRQoL of glaucoma patients from the perspective of cognition. Further evaluations are required to explore whether cognitive interventions that target illness perceptions can improve VRQoL in patients with glaucoma.

## Supplementary Information

**Additional file 1.** Results of the mediating effect analysis.

## Data Availability

The datasets generated or analysed during the current study are available from the corresponding author on reasonable request.

## Abbreviations

QoL: Quality of life
VRQoL: Vision-related quality of life
CSM: Common sense model
BIPQ: Brief illness perception questionnaire
GQL-15: Glaucoma quality of life-15
BCVA: Best-corrected visual acuity
VA: Vision acuity
VF: Visual field
MD: Mean defect
IOP: Intraocular pressure
logMAR: Logarithm of the minimum angle of resolution score
CCA: Canonical correlation analysis
