# Supplementary material for: Vision-related quality of life in patients with glaucoma: the role of illness perceptions
Source: Health Qual Life Outcomes. 2022 May 12;20:78. doi: 10.1186/s12955-022-01979-x (PMC9097073; doi:10.1186/s12955-022-01979-x)
Supplement: Supplementary file 1 — Additional file 1. Results of the mediating effect analysis. [file 12955_2022_1979_MOESM1_ESM.docx]

**Results of the mediating effect analysis**

Model diagram 1: Chronic comorbidities as the independent variable, vision-related quality of life (VRQoL) as the dependent variable, and illness perception as the mediating variable


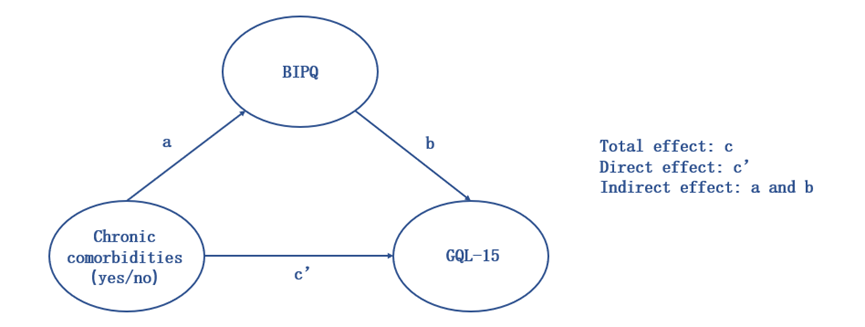


| **Effect of path** | **t** | **p value** |
| --- | --- | --- |
| c: total effect | 3.6485 | 0.0004 |
| a: chronic comorbidities→illness perceptions | 2.3459 | 0.0211 |
| b: illness perceptions→VRQoL | 3.5825 | 0.0005 |
| c’: chronic comorbidities→VRQoL | 2.9233 | 0.0043 |

| **Mediating effect of illness perceptions on chronic comorbidities and VRQoL** | | | | | |
| --- | --- | --- | --- | --- | --- |
|  | **Effect** | **Standard error** | **Lower limit**  **confidence interval** | **Upper limit**  **confidence interval** | **Effect proportion** |
| Indirect effect | 1.9315 | 1.0718 | 0.2699 | 4.3856 | 22.29% |
| Direct effect | 6.7356 | 2.3312 | 2.2494 | 11.3118 | 77.71% |
| Total effect | 8.6671 | 2.564 | 3.733 | 13.863 | 100% |

The mediating test was performed using the bootstrap method (number of bootstrap samples=5000).

Standard error refers to the standard error of indirect effects estimated by the bias-corrected bootstrap CI method; lower limit CI and upper limit CI refer to the lower limit and upper limit, respectively, of the 95% confidence interval.

The BIPQ measuring illness perceptions was entered as a potential mediator in the mediation model with chronic comorbidities as the independent variable and VRQoL as the dependent variable. The results showed that chronic comorbidities were directly related to VRQoL, and after entering the mediator variable into the model, the total effect and direct and indirect effects were all significant, suggesting that illness perceptions partially mediated the relationship between chronic comorbidities and VRQoL.

Model diagram 2: Type of glaucoma as the independent variable, vision-related quality of life (VRQoL) as the dependent variable, and illness perception as the mediating variable


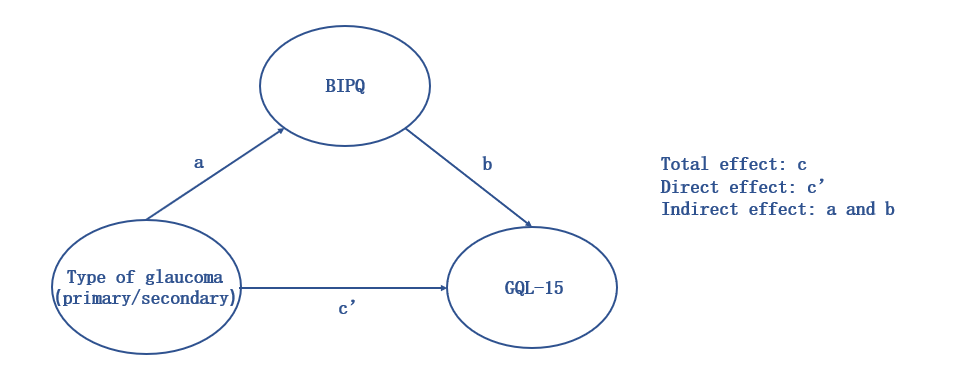


| **Effect of path** | **t** | **p value** |
| --- | --- | --- |
| c: total effect | 2.4148 | 0.0177 |
| a: type of glaucoma→illness perceptions | 3.6858 | 0.0004 |
| b: illness perceptions→VRQoL | 3.5551 | 0.0006 |
| c’: type of glaucoma→VRQoL | 1.1356 | 0.259 |

| **Mediating effect of illness perceptions on type of glaucoma and VRQoL** | | | | | | | | | |  |
| --- | --- | --- | --- | --- | --- | --- | --- | --- | --- | --- |
|  | **Effect** | | **Standard error** | | **Lower limit**  **confidence interval** | | **Upper limit**  **confidence interval** | | **Effect proportion** | |
| Indirect effect | 3.4314 | 1.6078 | | 0.9226 | | 7.1818 | | 52.55% | |  |
| Direct effect | 3.0988 | 2.9934 | | -2.6611 | | 8.9326 | | 47.45% | |  |
| Total effect | 6.5302 | 2.950 | | 0.823 | | 12.472 | | 100% | |  |

The mediating test was performed using the bootstrap method (number of bootstrap samples=5000).

Standard error refers to the standard error of indirect effects estimated by the bias-corrected bootstrap CI method; lower limit CI and upper limit CI refer to the lower limit and upper limit, respectively, of the 95% confidence interval.

The BIPQ measuring illness perceptions was entered as a potential mediator in the mediation model with the type of glaucoma as the independent variable and VRQoL as the dependent variable. The results showed that the total effect and indirect effect were both significant, whereas the direct effect of glaucoma type on VRQoL was not significant after entering the mediator variable into the model, suggesting that illness perceptions completely mediated the relationship between the type of glaucoma and VRQoL.
